# Intensified microbial sulfate reduction in the deep Dead Sea during the early Holocene Mediterranean sapropel 1 deposition

**DOI:** 10.1111/gbi.12493

**Published:** 2022-04-05

**Authors:** Elan J. Levy, Camille Thomas, Gilad Antler, Ittai Gavrieli, Alexandra V. Turchyn, Vincent Grossi, Daniel Ariztegui, Orit Sivan

**Affiliations:** ^1^ 26732 Department of Earth and Environmental Sciences Ben‐Gurion University of the Negev Beer Sheva Israel; ^2^ 61244 Geological Survey of Israel Jerusalem Israel; ^3^ Department of Climate Geochemistry Max Planck Institute for Chemistry Mainz Germany; ^4^ Department of Earth Sciences University of Geneva Geneva Switzerland; ^5^ The Interuniversity Institute for Marine Sciences in Eilat Eilat Israel; ^6^ Department of Earth Sciences University of Cambridge Cambridge UK; ^7^ Laboratoire de Géologie de Lyon Univ. Lyon 1 CNRS ENSL Villeurbanne France

**Keywords:** Dead Sea, Holocene, lipid biomarkers, microbial sulfate reduction, pore fluid, stable isotope composition of sulfate

## Abstract

The hypersaline Dead Sea and its sediments are natural laboratories for studying extremophile microorganism habitat response to environmental change. In modern times, increased freshwater runoff to the lake surface waters resulted in stratification and dilution of the upper water column followed by microbial blooms. However, whether these events facilitated a microbial response in the deep lake and sediments is obscure. Here we investigate archived evidence of microbial processes and changing regional hydroclimate conditions by reconstructing deep Dead Sea chemical compositions from pore fluid major ion concentration and stable S, O, and C isotopes, together with lipid biomarkers preserved in the hypersaline deep Dead Sea ICDP‐drilled core sediments dating to the early Holocene (ca. 10,000 years BP). Following a significant negative lake water balance resulting in salt layer deposits at the start of the Holocene, there was a general period of positive net water balance at 9500–8300 years BP. The pore fluid isotopic composition of sulfate exhibit evidence of intensified microbial sulfate reduction, where both $\delta^{34}S$ and $\delta^{18}O$ of sulfate show a sharp increase from estimated base values of 15.0‰ and 13.9‰ to 40.2‰ and 20.4‰, respectively, and a $\delta^{34}S$ vs. $\delta^{18}O$ slope of 0.26. The presence of the *n*‐C_17_ alkane biomarker in the sediments suggests an increase of cyanobacteria or phytoplankton contribution to the bulk organic matter that reached the deepest parts of the Dead Sea. Although hydrologically disconnected, both the Mediterranean Sea and the Dead Sea microbial ecosystems responded to increased freshwater runoff during the early Holocene, with the former depositing the organic‐rich sapropel 1 layer due to anoxic water column conditions. In the Dead Sea prolonged positive net water balance facilitated primary production and algal blooms in the upper waters and intensified microbial sulfate reduction in the hypolimnion and/or at the sediment–brine interface.

## 1 INTRODUCTION

**FIGURE 1:**
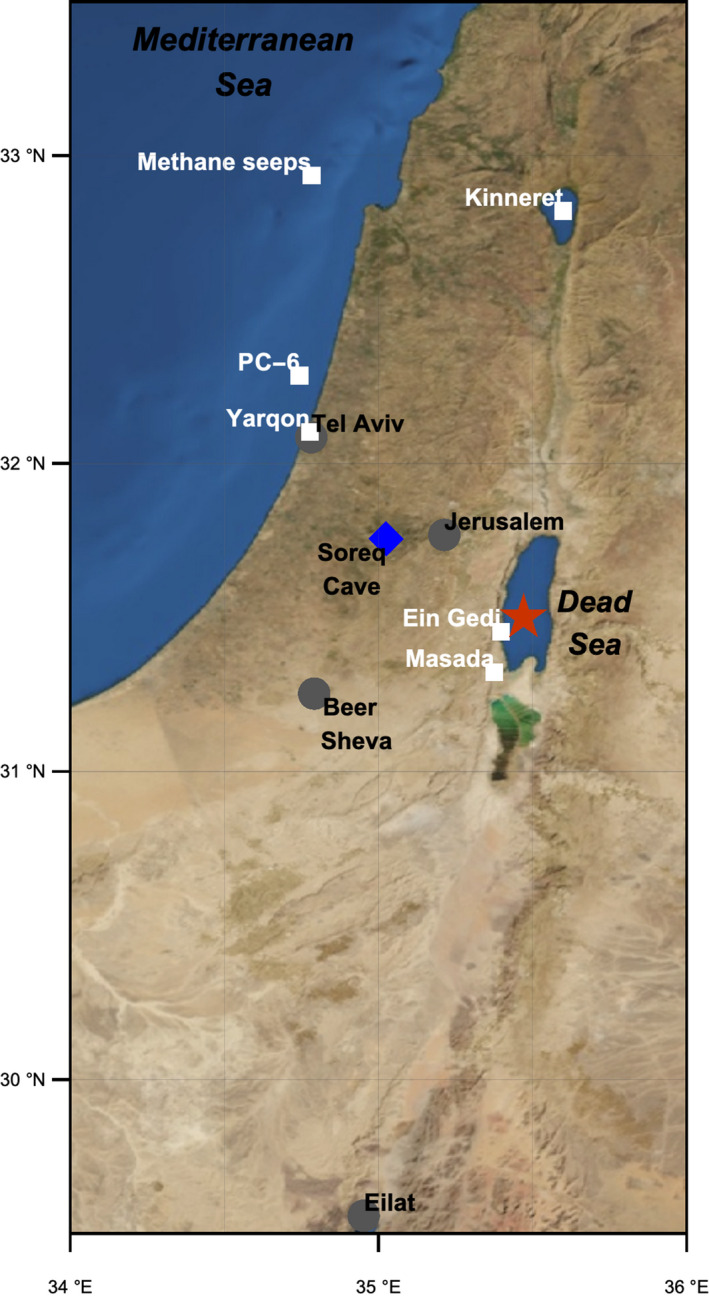
Map of the study region with the location of Dead Sea DSDDP core 5017‐1‐A (red star)

The Dead Sea is a terminal hypersaline lake (maximum water depth of ~300 m) located within a tectonically formed depression in an arid part of the Eastern Mediterranean (Figure 1). Before human intervention its water level reflected the water balance between freshwater influx, originating mostly from Mediterranean‐derived winter rainfall within the northern and central region of its 40,000‐km^2^ watershed, and evaporation (Enzel et al., 2003). Being highly concentrated (total dissolved solids: ~350 g L^−1^; density: ~1.24 Kg L^−1^), the Dead Sea is an extreme natural habitat, although specific populations of prokaryotes such as halophilic archaea and bacteria are adapted to this environment (Nissenbaum, 1975; Oren, 2010). The high brine density of the Dead Sea makes it extremely susceptible to stratification following heavy rainfall‐derived freshwater runoff events. Generally two conditions limit primary production in the Dead Sea: (1) high salinity and (2) phosphate nutrient availability (Oren et al., 2004; Oren & Shilo, 1985), both factors, which are impacted by freshwater runoff. Over the past few decades, spontaneous blooms of the unicellular primary producer green alga *Dunaliella parv*a directly followed by a dense, red‐pigmented halophilic archaea bloom have appeared twice, in the summer of 1980 and the spring of 1992, following particularly rainy winters with increased freshwater runoff that led to stratification of the Dead Sea water column and the formation of less saline surface waters (Oren, 1993; Oren et al., 1995).

Prior to 1979 overturn of the Dead Sea water column, the deep layer of the lake (hypolimnion) was anoxic and contained hydrogen sulfide (H_2_S) (Nissenbaum, 1975; Nissenbaum & Kaplan, 1976). The sulfur isotope values of the H_2_S (${\delta^{34}S}_{H_{2}S}$ = −19.6 to −21.7‰) were lower than that of the dissolved SO_4_^2−^ (${\delta^{34}S}_{{\mathrm{SO}}_{4}}$ = +15.9‰), and the H_2_S was suggested to have formed via microbial sulfate reduction (MSR) in the hypolimnion and/or in the underlying sediment interstitial water. In its generalized form, organoclastic‐type MSR involves the oxidation of an organic substrate coupled to the reduction of SO_4_^2−^ (Equation 1):

(1) $$2CH_{2}O+SO_{4}^{2‐}\to H_{2}S+2HCO_{3}^{‐}$$

where $CH_{2}O$ represents the mean of a variety of organic compounds in a subsurface marine setting (Arndt et al., 2013), and $H_{2}S$ the final reduced S product.

Whether modern deep‐dwelling MSR communities in the hypolimnion and/or deep sediments respond to transient periods of increased freshwater runoff is unknown, however, the sediment record may be used to shed light on whether such a connection occurred in the past. There have been a number of studies on MSR in the paleo‐Dead Sea using the distribution and stable isotope composition of S‐bearing minerals, such as gypsum (CaSO_4_·2H_2_O), pyrite (FeS_2_), elemental sulfur nodules (S_0_), greigite (Fe_3_O_4_), and mackinawite (FeS) (Bishop et al., 2013; Thomas et al., 2016; Torfstein et al., 2005, 2008; Torfstein & Turchyn, 2017). Investigation of MSR in paleolimnological studies has typically focused on the distribution and isotope composition of authigenic S‐bearing minerals. In modern environmental studies a more direct approach, such as measuring dissolved SO_4_^2−^ concentrations and its stable isotopic composition, is typically used (Avrahamov et al., 2014; Gavrieli et al., 2001).

During late 2010 and early 2011 the Dead Sea Deep Drilling Project (DSDDP) was undertaken as part of a joint international scientific effort and the International Continental Scientific Drilling Program (ICDP). Core 5017‐1‐A drilled from the deep Dead Sea (water column of ~300 m) comprises layered evaporites and clastic deposits spanning around 450‐m length and provides a near‐continuous depositional record covering >200 ka (Neugebauer et al., 2014; Torfstein et al., 2015). The core sediments allows for an investigation of the deep Dead Sea subsurface biosphere, an extreme habitat characterized by high salinity, toxic concentrations of divalent cations, and a lack of labile organic matter (OM) and nutrients (Bodaker et al., 2010; Ionescu et al., 2012; Oren, 1999). The analyses of biosignatures associated with this deep biosphere through the study of DNA and organominerals have been linked to changes in microbial diversity, microbial activity, and past paleoenvironmental and paleolimnological conditions (Thomas et al., 2014; Thomas et al., 2016).

The pore fluid samples extracted from the deep Dead Sea sediments in core 5017‐1‐A were shown to have been derived from deep lake brine trapped during sediment deposition (Levy et al., 2017). Subsurface advection and diffusion of pore fluid dissolved constituents were significantly hampered due to the abundant impermeable halite layers, high dynamic viscosity of hypersaline fluids, absence of bioturbation, and overall fast sedimentation rate (Levy et al., 2017). This is particularly true for the upper ~90m of core sediments corresponding to the Holocene. Pore fluid magnesium (Mg^2+^) and bromide (Br^−^) concentrations are conservative in core 5017‐1‐A (i.e., they did not significantly participate in chemical reactions and remained soluble; Supplementary Figure S1), and these ions provide a unique multimillennial scale record of dilution and concentration of the deep Dead Sea (Levy et al., 2017). The relatively high resolution of pore fluid compositional changes in the halite‐rich early Holocene sediments together with evidence of significant hydroclimate variability that occurred during this time makes it a suitable interval for reconstructing both microbial and hydroclimate processes, and for determining possible connections between them. During the early Holocene, there was a period of prolonged and relatively wet regional hydroclimate conditions which resulted in depositional changes in the Dead Sea (Neugebauer et al., 2017). In this study, we investigate the 5017‐1‐A core sediment distribution and pore fluid concentrations, combined with pore fluid stable S, O, and C isotope ratios, lipid biomarkers, and organic C isotope compositions to reconstruct regional hydroclimate changes, microbial processes, and microbial sulfate reduction activity in the early Holocene Dead Sea.

## 2 MATERIALS AND METHODS

### 2.1 DSDDP 5017‐1‐A Holocene chronology

**FIGURE 2:**
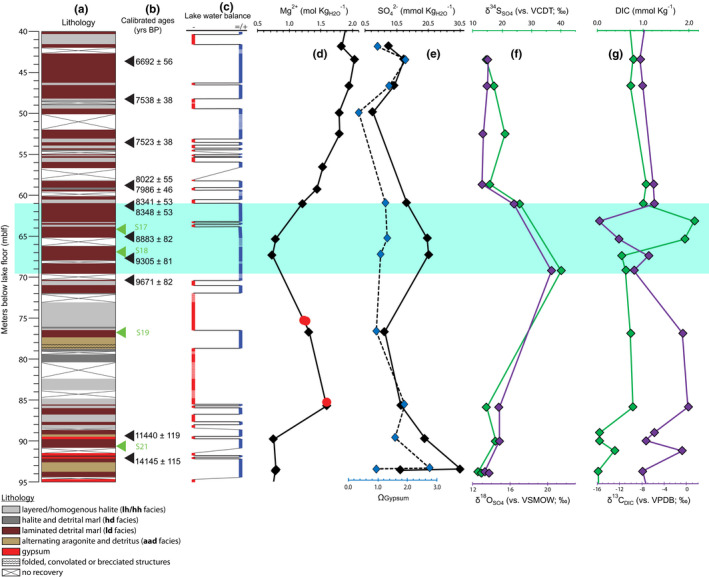
Core 5017‐1‐A depth profiles. The interval of interest in the early Holocene is highlighted in turquoise. (a) 5017‐1‐A lithology adapted from Neugebauer et al. (2014); (b) calibrated radiocarbon ages (Neugebauer et al., 2014 and Kitagawa et al., 2017). Green triangles indicate depths where lipid biomarkers were analyzed for this study (samples S17, S18, S19, S21; Figure 3); (c) Negative vs. positive/steady‐state lake water balance running curve based on general lithology; (d) Mg^2+^ concentrations (mol·Kg_H2O_^−1^) in pore fluids (black diamonds) (after Levy et al., 2017) and fluid inclusions from primary halite (red circles) (after Kiro et al., 2017); (e) SO_4_^2−^ concentrations (mmol Kg_H2O_^−1^) (black diamonds) and the degree of saturation for gypsum (Ω_Gypsum_) (blue diamonds) (after Levy et al., 2019); (f) ${\delta^{34}S}_{{\mathrm{SO}}_{4}}$ values (‰; vs. VCDT; in green, top *x*‐axis) and ${\delta^{18}O}_{{\mathrm{SO}}_{4}}$ values (‰; vs. VSMOW; in purple, bottom *x*‐axis) (after Levy et al., 2019); (g) Dissolved inorganic carbon concentration (DIC; mmol Kg^−1^) (in green, top *x*‐axis), and ${\delta^{13}C}_{\mathrm{DIC}}$ values (‰; vs. VPDB) (in purple, bottom *x*‐axis)

The 456‐meter sedimentary core 5017‐1‐A was extracted from the deepest part of the lake at 300‐m water column depth (~720 m below mean sea level) at 31°30′28.98″N, 35°28′15.60″E. The chronology of the investigated sediment sequence for Holocene sediments (Figure 2a) is based on a linear interpolation of calibrated radiocarbon ages derived from terrestrial plant remains from site 5017‐1 (Kitagawa et al., 2017; Neugebauer et al., 2014; Figure 2b). The calibration was done using the IntCal13 calibration dataset (Reimer et al., 2013) within the oxcal 4.3 software.

### 2.2 Binary net lake water balance curve

A binary net lake water balance curve for the Holocene was drawn based on the appearance of indicative sediment facies in core 5017‐1‐A (Neugebauer et al., 2014; Torfstein et al., 2015; Figure 2c) as the thickness of evaporite deposits may not reflect the relative magnitude of net water balance changes. Layered halite sediments (*lh* and *hh* facies in Neugebauer et al., 2014) and gypsum are interpreted as being formed following negative water balance based on the assumption that decreases in lake level leading to increased salinity is the dominant factor for precipitation of these layered evaporite units. The laminated detrital marl and alternating aragonite and detritus facies (*ld* and *aad* facies, respectively) are assumed to have formed during times of either steady‐state balance or positive net water balance. Dashed lines are drawn at sediment interval depths of no sediment recovery (Figure 2c).

### 2.3 Pore fluid extraction, handling, and analyses

A description of sediment handling for core catcher material, taken during the drilling campaign of winter 2010–2011 and core section sediments, taken during July 2012, including pore fluid extraction methods, post‐extraction treatment, and measurement of major cation and anion concentrations are given by Levy et al. (2017, 2019). Following pore fluid extraction, subsamples for dissolved inorganic carbon (DIC) concentration and stable isotope analyses were immediately transferred into 20‐mL prepoisoned (HgCl_2_ powder) airtight syringes to terminate the microbial activity. About 0.2 ml of each sample was injected into a He‐preflushed vial containing H_3_PO_4_ for the headspace measurements of ${\delta^{13}C}_{\mathrm{DIC}}$ by isotope ratio mass spectrometry (IRMS, DeltaV Advantage; Thermo) with a precision of ±0.1‰, and DIC concentrations based on peak volume (±0.2 mm).

Pore fluid sulfate was precipitated as barium sulfate (barite) using a saturated barium chloride solution. The barite was then washed once with 6‐N HCl and twice with deionized water. For the analysis of ${\delta^{18}O}_{{\mathrm{SO}}_{4}}$, barite was pyrolyzed at 1450°C in a temperature conversion element analyzer (TC/EA), producing carbon monoxide, which was measured in a GS‐IRMS (Thermo Finnegan Delta V Plus, at the Godwin Laboratory, University of Cambridge). To analyze ${\delta^{34}S}_{{\mathrm{SO}}_{4}}$, barite was combusted at 1030 °C in a flash element analyzer (EA), and the resulting sulfur dioxide (SO_2_) was measured on a GS‐IRMS (Thermo Finnegan Delta V Plus Godwin Laboratory, University of Cambridge). Analyses of ${\delta^{18}O}_{{\mathrm{SO}}_{4}}$ were conducted in replicates (*n* = 3–5), and the standard deviation of these replicate analyses is reported (~0.4‰ 1σ). The error for ${\delta^{34}S}_{{\mathrm{SO}}_{4}}$ was determined using the standard deviation of multiple standards (*n* = 6) at the beginning and the end of each run (~0.3‰ 1σ). Measurements of ${\delta^{18}O}_{{\mathrm{SO}}_{4}}$ were corrected to NBS 127, IAEA‐SO‐6, and IAEA‐SO‐5 (8.6‰, −11.35‰, and 12.1‰, respectively). Measurements of ${\delta^{34}S}_{{\mathrm{SO}}_{4}}$ were corrected to NBS 127, IAEA‐SO‐6, IAEA‐SO‐5, and IAEA‐S‐3 (20.3‰, −34.1‰, 0.5‰, and −32.4‰, respectively). The ${\delta^{18}O}_{{\mathrm{SO}}_{4}}$ is reported relative to the Vienna Standard Mean Ocean Water (VSMOW), and ${\delta^{34}S}_{{\mathrm{SO}}_{4}}$ is reported with respect to Vienna Canyon Diablo Troilite (VCDT) (Antler et al., 2013).

The saturation state of gypsum ($\Omega_{{\mathrm{CaSO}}_{4}∙2H_{2}O}$; Equation 2) was calculated using PHREEQC© version 3.1.2–8538 applying the “Pitzer” approach at 25°C (Parkhurst & Appelo, 1999; Pitzer, 1973; Reznik et al., 2009).

(2) $$\Omega_{{\mathrm{CaSO}}_{4}∙2H_{2}O}=\frac{I.A.P.}{K_{{\mathrm{CaSO}}_{4}∙2H_{2}O}}$$

where I.A.P. and $K_{{\mathrm{CaSO}}_{4}∙2H_{2}O}$ denote ion activity product and the gypsum solubility product, respectively.

### 2.4 Quantification of MSR

An adapted Rayleigh distillation equation for stable isotope evolution was used to estimate the fraction (f) of residual SO_4_^2−^ remaining following MSR (Equation 3):

(3) $$f={\left(\frac{{\delta^{34}S}_{{\mathrm{SO}}_{4},max}\;+\;1000}{{\delta^{34}S}_{{\mathrm{SO}}_{4},i}\;+\;1000}\right)}^{\frac{1}{\alpha ‐1}}$$

where ${\delta^{34}S}_{{\mathrm{SO}}_{4},i}$ is the stable sulfur isotope value for SO_4_^2−^ prior to MSR, which we estimate to be 15.0‰ (mean ${\delta^{34}S}_{{\mathrm{SO}}_{4}}$ values from early and middle Holocene pore fluids excluding the interval of interest; see section 3.2), ${\delta^{34}S}_{{\mathrm{SO}}_{4},max}$ is the value following MSR that is 40.2‰ and α is the fractionation factor during MSR. An isotope fractionation (ε) range between 22‰ and 67‰ was used (Deusner et al., 2014). Following the calculation of f, the pre‐MSR SO_4_^2−^ concentrations and degree of saturation with respect to gypsum were estimated.

Additionally, we use a stoichiometric ratio of organic C:SO_4_ of 2:1 based on the generalized pathway of MSR (i.e., Equation 1) to obtain a first‐order approximation for the amount of organic carbon oxidized (weight %; relative to sediment) by the calculated reduced SO_4_^2−^ (i.e., absolute values corrected for f from Equation 3) (Equation 4):

(4) $$C_{\text{org}\text{.ox}.}=\frac{2\left(\frac{SO_{4\;PF}^{\;2‐}}{f}‐SO_{4\;PF}^{\;2‐}\right)\times \left(1‐\Phi \right)\times M\text{.W}._{CH_{2}O}\times 10^{‐3}}{\rho }$$

where ${{{\mathrm{SO}}_{4}}^{2‐}}_{\mathrm{PF}}$ is the respective pore fluid concentration in m, Φ is the estimated porosity (i.e., L_PF_ · L_Total_^−1^; Φ = 0.4; Levy et al., 2017), ${M.W.}_{CH_{2}O}$ is the molecular weight for the average organic substrate in organoclastic MSR (Equation 1; $CH_{2}O$—30.031 g m), 10^−3^ converts L to cm^3^, and ρ is a first‐order estimate for the bulk density of the sediment (~2 g cm^−3^).

### 2.5 Calculating *δ* ^13^ *C* of added DIC

The observed changes in DIC concentrations and respective ${\delta^{13}C}_{\mathrm{DIC}}$ in measured pore fluid compositions were used to calculate the addition of DIC derived from OM oxidation (between two corresponding depths: initial (*i*) to final (*f*)) using the following mass balance equation for a closed system (Equation 5):

(5) $$\delta^{13}C=\frac{\left[\left({\mathrm{DIC}}_{f}\times {\delta^{13}C}_{DIC,f}\right)‐\left({\mathrm{DIC}}_{i}\times {\delta^{13}C}_{DIC,\;i}\right)\right]}{\left({\mathrm{DIC}}_{f}‐\;{\mathrm{DIC}}_{i}\right)}$$

### 2.6 Lipid biomarker extraction and analysis

**FIGURE 3:**
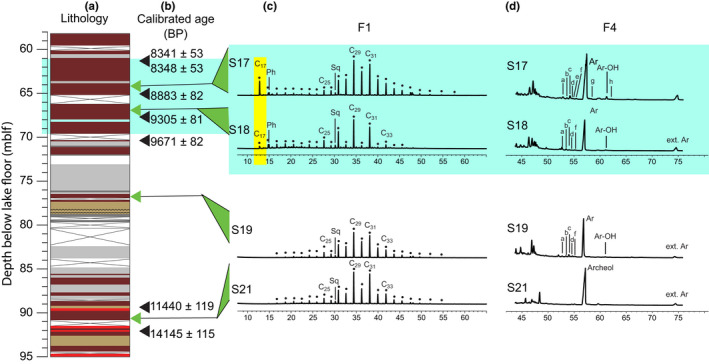
Lipid biomarker distributions from selected samples. The interval of interest in the early Holocene is highlighted in turquoise. (a) 5017‐1‐A lithology (Neugebauer et al., 2014; see Figure 2 for details); (b) calibrated radiocarbon ages (Kitagawa et al., 2017; Neugebauer et al., 2014). Depths where lipid biomarkers were analyzed are highlighted in green; (c) selective mass chromatograms (m/z = 57) of hydrocarbon fractions (F1); (d) partial total ion chromatograms of alcohol fractions (F4). Black dots and Cx, linear alkanes with x carbon atoms; Ph, phytane; Sq; squalane; Ar, archaeol; Ar‐OH, *sn*‐2‐hydroxyarchaeol; a‐g, nonisoprenoid macrocyclic glycerol diethers; h, macrocyclic archaeol

Four sediment samples between 65 and 91 meters below the lake floor (mblf) were analyzed for their lipid composition (S17, S18, S19, and S21; Figure 3). Samples were freeze‐dried, ground, and extracted through sonication (methanol (MeOH) twice, MeOH/dichloromethane (DCM) (1:1) twice, and DCM three times). Sulfur was removed by activated copper. Precipitates were filtered out and lipids separated into five fractions by chromatography over a deactivated column of silica gel. Fraction F1 was eluted with hexane/DCM (9:1), F2 with hexane/DCM (1:1), F3 with DCM, F4 with ethyl acetate, and F5 with MeOH. Fraction F4 was silylated with pyridine/bis(trimethylsilyl) trifluoroacetamide (BSTFA) 1:1 (v/v). Only fractions F1 and F4 are presented in this article. Gas chromatography–mass spectrometry analyses (GC‐MS) were performed using an HP 6890 Series Plus gas chromatograph equipped with a cool on‐column injector and coupled to an Agilent 5975C (VL MSD) mass spectrometer. The GC was equipped with an HP5 column (30 m × 0.25 mm × 0.25 µm, RESTEK). Samples were injected at 60°C (held for 30 s) before oven temperature was increased to 130°C at 20°C min^−1^, then to 250°C (5°C min^−1^) and 300°C (3°C min^−1^, held for 45 min). Compound‐specific carbon isotope ($\delta^{13}C$) analyses were done using an HP7890B GC coupled to an Isoprime visION isotope ratio mass spectrometer via a GC‐5 combustion interface operating at 870°C. The GC was equipped with a BPX5 column (30 m × 0.25 mm × 0.10 µm, SGE Analytical Science) and a cool on‐column injector, and the oven temperature was programmed for GC‐MS analyses. The B4 standard mixture (Arndt Schimmelmann, Indiana University, USA) was used to externally calibrate compound‐specific ^13^C values, and ^13^C values of alcohols were corrected from the BSTFA‐derivatizing agent (Thomas, Grossi, et al., 2019).

## 3 RESULTS AND DISCUSSION

### 3.1 Dead Sea early Holocene sediment interval

The Holocene interval of 5017‐1‐A is 88 m. The early Holocene “interval of interest” (a term we will use hereafter) is a depth interval located between 70 to 61 mblf and dates to ca. 9500–8300 years BP. It is composed of a thick section of laminated detrital marl (*ld*) facies punctuated by sparse‐layered halite at 64/63 mblf (Figure 2a) (Neugebauer et al., 2014). The interval of interest overlay sediments deposited at the onset of the Holocene at 88 to 70 mblf (from ca. 11,000 to 9700 years BP) comprising layered/homogenous halite (*lh*/*hh* facies) interbedded with laminated detritus (*ld*) and some alternating aragonite and detritus (*aad*). The most dilute conservative Mg^2+^ concentrations of 0.73 and 0.78 mol·Kg_H2O_^−1^ are found in the interval of interest at 65.4 and 67.4 mblf, respectively (9000 ± 250 years BP) (Figure 2d; Levy et al., 2017). From the base of the section up, there is an increase of SO_4_^2−^ concentrations from 6.7 mmol Kg_H2O_^−1^ at 77 mblf to 20.7 mmol Kg_H2O_^−1^ (Figure 2e). Maximum values of ${\delta^{34}S}_{{\mathrm{SO}}_{4}}$ (40.2‰) and ${\delta^{18}O}_{{\mathrm{SO}}_{4}}$ (20.4‰) are also observed (Figure 2f). In the upper part of the interval of interest there is a maximum peak of DIC concentration of 2.1 mmol Kg^−1^ and a minimum ${\delta^{13}C}_{\mathrm{DIC}}$ value of −15.6‰ (Figure 2g; green line for DIC and purple line for ${\delta^{13}C}_{\mathrm{DIC}}$). The lipid biomarker fractions F1 are dominated by *n*‐alkanes, with a general odd‐over‐even carbon number predominance and centered on C_29_ and C_31_ *n*‐alkanes (Figure 3c). In the interval of interest (samples S17 and S18) F1 fractions also contain significant amounts of *n*‐C_17_ alkane and phytane (highlighted in yellow; Figure 3c). The main features of the F4 fractions are the strong proportions of archaeol and extended archaeol (Figure 3d). Contributions of varied nonisoprenoid macrocyclic glycerol diethers and hydroxyarchaeol are also evident in samples S17, S18, and S19 (Figure 3d; Supplementary Figure S2 and S3). The $\delta^{13}C$ of total lipid extracts in the interval 61.9 to 89.3 mblf ranged between −36.8‰ (±0.4) and −38.3‰ (±0.3) (Table 1). The $\delta^{13}C$ of archaeol ranges between −25.2‰ (±0.3) and −30.8‰ (±0.3) with slightly heavier values ranging between −27.8‰ and −25.2‰ in the interval of interest (Table 1).

**TABLE 1:** Measured δ^13^C values of the total lipid extracts and archaeol of selected samples from early Holocene sediments in the Dead Sea 5017 sediment core

Sample ID#	Depth (mblf)	Core section ID#	δ¹³C‐Total lipid extracts (‰) and SD		δ¹³C‐Archaeol (‰) and SD	
OG30	61.86	5017‐1A‐31‐2	−37.9	0.5	−25.2	0.3
OG32	64.83	5017‐1A‐32‐2	−36.8	0.4	−25.8	0.0
OG34	69.19	5017‐1A‐34‐1	−37.1	0.7	−27.8	0.0
OG38	77.25	5017‐1A‐38‐1	−36.8	0.3	−29.8	0.2
OG43	89.33	5017‐1A‐43‐1	−38.3	0.3	−30.8	0.3

The vertical distribution of halite and gypsum vs. detritus‐bearing sediments together with a binary net‐water‐balance curve (Figure 2c; see methods) reflects the net‐water‐balance changes that occurred during the early Holocene. With the additional information provided by conservative ion concentrations from the pore fluids and fluid inclusions from primary halite (i.e., halite precipitated from the lake water column), millennial‐scale net‐water balance changes in the hypolimnion can be quantified (Levy et al., 2017).

Halite (NaCl) evaporite layers dating to the start of the Holocene deposited as a result of a sequence of lake level drops (negative water balance), which began at the end of the last glacial period (Torfstein et al., 2015). The lithology changes are supported by increased Mg^2+^ ion concentrations in pore fluids from 0.75 mol Kg_H2O_^−1^ below the halite (pre‐Holocene) at 89.8 mblf (ca. 12,500 years BP) to 1.59 mol Kg_H2O_^−1^ at 85.7 mblf (ca. 11,000 years BP), and by a concentration of 1.61 mol·Kg_H2O_^−1^ in a fluid inclusion within primary halite at 85.4 mblf (Figure 2d) (Kiro et al., 2017; Levy et al., 2017). A similarity between the independently measured pore fluid and primary fluid inclusion conservative concentrations not only supports the use of both measurements for the reconstruction of deep lake compositions but also suggests that pore fluid in the Lisan Fm. sediments below (i.e., > 90 mblf) did not penetrate through the underlying thick layered halite section following sediment burial.

Beginning at ~79 mblf, the presence of *ld* and *aad* type sediments suggests a general shift from a negative to a positive lake water balance. This is corroborated by decreased pore fluid and primary halite fluid inclusion conservative ion concentrations (Figure 2d): Pore fluid Mg^2+^ of 1.31 mol Kg_H2O_^−1^ at 76.8 mblf (ca. 10,100 years BP) is more dilute than at 85.7 mblf with 1.59 mol Kg_H2O_^−1^. Two fluid inclusions from primary halite above, at 75 mblf, are similarly dilute at 1.24 and 1.28 mol Kg_H2O_^−1^, respectively (Figure 2d). This upward dilution trend continues into the overlying *ld* dominated interval of interest highlighted in turquoise (Figure 2).

The appearance of laminated detrital marl (*ld*) facies together with dilute conservative ion concentrations suggests intermittent periods of positive water balance prior to the reprecipitation of halite at ca. 8200 years ago in the Dead Sea. The re‐precipitation of halite at a higher lake level following a period of low levels requires addition of Na^+^ and Cl^−^ to the lake solute inventory accumulating during the intermittent period of lake dilution. The increase in the Na/Cl ratio in both pore fluids (from ~86 to 65 mblf) and in halite fluid inclusions (Supplementary Figure S1) together suggest that halite dissolution likely occurred coeval with lake dilution as emphasized by the Mg^2+^ (Kiro et al., 2017; Levy et al., 2017, 2018).

### 3.2 Sulfate, ${\delta^{34}S}_{{SO}_{4}}$ **and** ${\delta^{18}O}_{{SO}_{4}}$ **controls**

Early Holocene pore fluid SO_4_^2−^ concentration trends appear to somewhat mirror concentrations of Mg^2+^, i.e., increasing (or decreasing) SO_4_^2−^ with decreasing (or increasing) Mg^2+^ (Figure 2d and e—black diamonds; Levy et al., 2017, 2019). The diluted Mg^2+^ in the interval of interest is indicative of lake dilution, while the nonconservative SO_4_^2−^ on the other hand is a record of solute inventory enrichment. High SO_4_^2−^ concentrations and gypsum saturated and supersaturated conditions (1.0 ≤ $\Omega_{{\mathrm{CaSO}}_{4}∙2H_{2}O}$ ≤ 1.3) are characteristic of these pore fluid samples (Figure 2e—blue diamonds). What sources contributed to the observed SO_4_^2−^ enrichment?

Several models have been proposed for the sources of SO_4_^2−^ to the modern Dead Sea and its late Pleistocene Lake Lisan precursor. Presently, the freshwater sources to the gypsum‐supersaturated Dead Sea carry substantially lower SO_4_^2−^ concentrations than in the Dead Sea itself (range of 0.04 mm in Golan basaltic springs to 1.24 mm in Wadi Hasa) (Torfstein et al., 2005). Gypsum supersaturation is a typical feature of Dead Sea–type “calcium‐chloride” brines (Gavrieli et al., 2001), and is a metastable state whereby kinetic factors in the solution and an absence of suitable nuclei prevent mineralization. In the modern Dead Sea, the ongoing negative water balance throughout the later part of the 20th century resulted in brine concentration and increasing degree of supersaturation with respect to gypsum ($\Omega_{{\mathrm{CaSO}}_{4}∙2H_{2}O}$ = 1.42 ± 0.11 during 2008) (Reznik et al., 2009). In the past, Dead Sea surface water dilution resulted in lowered Ca^2+^ concentrations, which then allowed the inflowing SO_4_^2−^ to accumulate in the terminal lake via a mechanism of influx and evaporation over time, thus resulting in increasing SO_4_^2−^ concentrations (Gavrieli et al., 2001). Precipitation of gypsum in the epilimnion and sinking of these mineral particles results in the transfer and enrichment of SO_4_^2−^ (via dissolution) in the diluted hypolimnion in the stratified water column (i.e., “sulfur pump model”; Gavrieli et al., 2001; Torfstein et al., 2005).

Saline springs on the western side of the Dead Sea, which have concentrations in excess of modern‐day brine (~10 mm), may also contribute sulfate to the Dead Sea (Gavrieli et al., 2001; Torfstein et al., 2005; Weber et al., 2022). Springs such as the present‐day Ein Qedem were activated following the regional aridity and associated lake level drops at the end of the late Pleistocene (Weber et al., 2018) and may have been active at the start of the Holocene (Weber et al., 2022). An additional SO_4_^2−^ source and enrichment mechanism for the hypolimnion is the dissolution of calcium sulfate minerals in the upper waters (i.e., at the Mt. Sedom salt diapir) and transfer via gravity‐driven brine flows internally in the Dead Sea, a mechanism that was suggested for the last glacial period (Levy et al., 2019). Other potential sources include SO_4_^2−^ enrichment occurring via in situ gypsum dissolution, in the subsurface or early Holocene water column, or reduced mineral bound S oxidation. Gypsum dissolution may occur directly following MSR in the hypolimnion or the subsurface sediments provided that SO_4_^2−^ concentrations decrease to the extent that the solution becomes undersaturated with respect to gypsum (Gavrieli et al., 2001; Torfstein et al., 2005).

Unlike the SO_4_^2−^ concentrations, the pore fluid ${\delta^{34}S}_{{\mathrm{SO}}_{4}}$ and ${\delta^{18}O}_{{\mathrm{SO}}_{4}}$ provide an archive of MSR. The interval of interest has relatively ^34^S and ^18^O‐enriched SO_4_^2−^ (maximum values of ${\delta^{34}S}_{{\mathrm{SO}}_{4}}$ = 40.2‰ and ${\delta^{18}O}_{{\mathrm{SO}}_{4}}$ = 20.4‰; Figure 2f). Mean ${\delta^{34}S}_{{\mathrm{SO}}_{4}}$ and ${\delta^{18}O}_{{\mathrm{SO}}_{4}}$ values from early and middle Holocene pore fluids, excluding this interval, have values of 15.0‰ (±2.1) and 13.9‰ (±0.7), respectively. These averages are only slightly higher relative to measurements of ${\delta^{34}S}_{{\mathrm{SO}}_{4}}$ = 14.1‰ (±0.3) and ${\delta^{18}O}_{{\mathrm{SO}}_{4}}$ = 11.4‰ (±0.3) for Dead Sea upper waters (<100 m) sampled in 2013 (Levy et al., 2019). During dissimilatory MSR, there is a microbial preference to utilize the lighter and abundant ^32^S and ^16^O isotopes from the SO_4_^2−^ reservoir resulting in ^34^S‐depleted $H_{2}S$ product, along with ^34^S‐ and ^18^O‐enriched residual SO_4_^2−^. Although the ${\delta^{34}S}_{{\mathrm{SO}}_{4}}$ and ${\delta^{18}O}_{{\mathrm{SO}}_{4}}$ in the interval of interest appears to reflect MSR (Figure 2f), given the uniqueness of the hypersaline Dead Sea environment, other processes must also be considered such as gypsum precipitation and gypsum dissolution, and changes in the source of SO_4_^2−^ with differing stable isotopic compositions. The difference between the measured maximum and mean S isotope composition are +25‰ for ${\delta^{34}S}_{{\mathrm{SO}}_{4}}$, and while gypsum precipitation does result in some positive sulfur isotope fractionation (ε), it is typically only between +1‰ and +2‰ (e.g., Raab & Spiro, 1991) and cannot explain the observed magnitude of isotopic difference. Furthermore, there are no gypsum layers in the Dead Sea sedimentary record in core 5017‐1‐A, at the marginal terraces, nor at Mt. Sedom, with comparably high ${\delta^{34}S}_{{\mathrm{SO}}_{4}}$. Thus gypsum dissolution alone cannot explain the observed ${\delta^{34}S}_{{\mathrm{SO}}_{4}}$ and ${\delta^{18}O}_{{\mathrm{SO}}_{4}}$ values (Levy et al., 2019; Torfstein & Turchyn, 2017). Finally, there are no known natural water sources to the modern Dead Sea with such ^34^S‐enriched SO_4_^2−^ isotope composition (Torfstein et al., 2005). The observed increases in the estimated mean base values of ${\delta^{34}S}_{{\mathrm{SO}}_{4}}$ = 15.0‰ and ${\delta^{18}O}_{{\mathrm{SO}}_{4}}\;$= 13.9‰ to maximum values of 40.2‰ and 20.4‰, respectively, can thus be assumed to have resulted from MSR (Figure 2f).

We calculated the amount of SO_4_^2−^ reduced using the range of S isotope fractionations (*ε* ^34^ *S*) of +22‰ and +69‰, derived from incubation experiments with various CH_4_ concentrations (Duesner et al., 2014). This range mostly encompasses the range of sulfur isotope fractionation associated with both organoclastic sulfate reduction and sulfate reduction coupled to methane oxidation (AOM). Assuming closed system and Rayleigh‐type distillation isotope fractionation (Equation 3), we estimate that between ~66 and 30% of the SO_4_^2−^ had thus been reduced (i.e., f = 0.33 and 0.70). Pore fluid SO_4_^2−^ concentrations in the interval of interest remain at or slightly above saturation with respect to gypsum (Figure 2e). This would suggest that one of the following mechanisms may have occurred: (a) prior to MSR, pore fluid was initially at a higher degree of supersaturation with respect to gypsum (mechanism A), (b) the MSR was accompanied by gypsum dissolution (mechanism B), or (c) a combination of both mechanisms occurred (i.e., A followed by or alongside B). Exploring the first mechanism (A), we estimate that the initial solution would be at $\Omega_{{\mathrm{CaSO}}_{4}∙2H_{2}O}$ = 3.2 to 1.5, respectively, and 38 to 8 mM of SO_4_^2−^ would have been reduced to bring the pore fluid to saturation ($\Omega_{{\mathrm{CaSO}}_{4}∙2H_{2}O}$ ~ 1), respectively (Equation 2). Assuming SO_4_^2−^ is reduced primarily by coupling with anaerobic oxidation of methane (AOM), this would amount to equivalent oxidation of CH_4_. Alternatively, assuming a molar ratio of S:C of 1:2 for MSR (Equation 1), the amount of SO_4_^2−^ reduction would correspond to 0.001 to 0.01% (total sedimentary weight percent) organic C being utilized (see methods section 2.4; Equation 4). Had gypsum dissolution occurred coeval with MSR (mechanism B), a larger amount of SO_4_^2−^ would have been reduced (Gavrieli et al., 2001; Torfstein et al., 2005) and facilitated by more OM oxidation.

**FIGURE 4:**
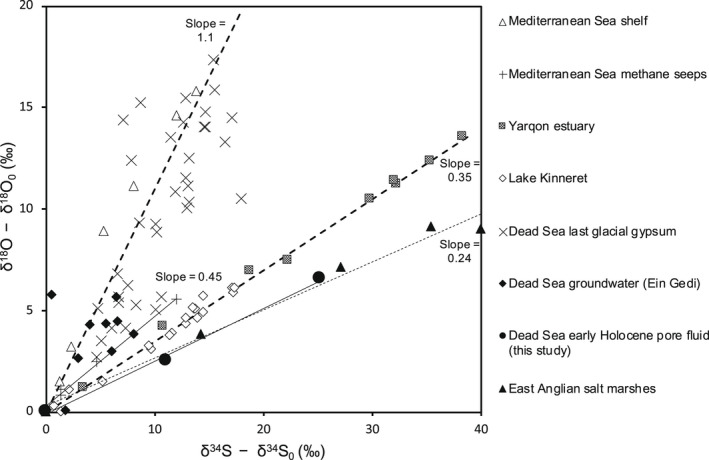
(${\delta^{18}O}_{{\mathrm{SO}}_{4}}$ ‐ ${\delta^{18}O}_{{\mathrm{SO}}_{4},0}$) vs. (${\delta^{34}S}_{{\mathrm{SO}}_{4}}$ ‐ ${\delta^{34}S}_{{\mathrm{SO}}_{4},0}$) plot for a range of SO_4_^2−^ sources (marine, lacustrine, groundwater and subsurface pore fluids), and Dead Sea gypsum deposits. Values for ${\delta^{34}S}_{{\mathrm{SO}}_{4},0}$ and ${\delta^{18}O}_{{\mathrm{SO}}_{4},0}$ are the lowest within the individual datasets unless stated otherwise. Datasets presented: shelf sediments from site PC‐6 in the Mediterranean Sea (open triangles) with ${\delta^{34}S}_{{\mathrm{SO}}_{4},0}$ = 20.2‰ and ${\delta^{18}O}_{{\mathrm{SO}}_{4},0}$ = 9.4‰ (Rubin‐Blum et al., 2014); primary gypsum layers deposited in and around the Dead Sea dating back to the last glacial period (crosses) with estimated initial Lake Lisan SO_4_^2−^ values of ${\delta^{34}S}_{{\mathrm{SO}}_{4},0}\;$= 10‰ and ${\delta^{18}O}_{{\mathrm{SO}}_{4},0}$ = 10‰ (Torfstein & Turchyn, 2017); groundwater at Ein Gedi along the Dead Sea coast (black diamonds) with ${\delta^{34}S}_{{\mathrm{SO}}_{4},0}$ = 15.9‰ and ${\delta^{18}O}_{{\mathrm{SO}}_{4},0}\;$= 13.7‰ (Avrahamov et al., 2014); Eastern Mediterranean site NA‐80 methane cold‐seeps (plus signs) with ${\delta^{34}S}_{{\mathrm{SO}}_{4},0}$ = 20.3‰ and ${\delta^{18}O}_{{\mathrm{SO}}_{4},0}$ = 8.6‰ (Antler et al., 2015); Lake Kinneret stratified water column (open diamonds; LK4 expedition 10/2012) with ${\delta^{34}S}_{{\mathrm{SO}}_{4},0}$ = 12.6‰ and ${\delta^{18}O}_{{\mathrm{SO}}_{4},0}$ = 11.3‰ (Knossow et al., 2015); Yarqon estuary (gray squares) with ${\delta^{34}S}_{{\mathrm{SO}}_{4},0}$ = 21.1‰ and ${\delta^{18}O}_{{\mathrm{SO}}_{4},0}$ = 9.5‰ (Antler et al., 2013); and East Anglian salt marshes (black triangles) with ${\delta^{34}S}_{{\mathrm{SO}}_{4},0}$= 34.3‰ and ${\delta^{18}O}_{{\mathrm{SO}}_{4},0}$ = 16.1‰ (Antler et al., 2019). Early Holocene Dead Sea pore fluid samples from the defined interval of interest in this study with ${\delta^{34}S}_{{\mathrm{SO}}_{4},0}$ = 15.0‰ and ${\delta^{18}O}_{{\mathrm{SO}}_{4},0}$ = 13.9‰ of averaged non‐MSR pore fluid data from early/middle Holocene (see also Supplementary Figure S2).

The metabolism of MSR can also be explored using ${\delta^{34}S}_{{\mathrm{SO}}_{4}}$ and ${\delta^{18}O}_{{\mathrm{SO}}_{4}}$. During MSR, intracellular intermediate species are produced within the cytoplasmic membrane (e.g., Adenosine 5’‐phosphosulfate, sulfite—SO_3_^2−^, S^2+^) and there is an exchange of oxygen isotopes with water before the final sulfide product is released to the surrounding aqueous anoxic environment (Rees, 1973). As some of these intermediates are re‐oxidized back to SO_4_^2−^, they influence the relative evolution of ${\delta^{34}S}_{{\mathrm{SO}}_{4}}$ versus ${\delta^{18}O}_{{\mathrm{SO}}_{4}}$ where more re‐oxidation causes a larger change in ${\delta^{18}O}_{{\mathrm{SO}}_{4}}$ relative to ${\delta^{34}S}_{{\mathrm{SO}}_{4}}$. Changes in the ${\delta^{34}S}_{{\mathrm{SO}}_{4}}$ and ${\delta^{18}O}_{{\mathrm{SO}}_{4}}$ have been suggested to depend on the magnitude of re‐oxidation of intracellular intermediates and to be proportional to the overall rate of MSR (Antler et al., 2013; Böttcher et al., 1998; Brunner et al., 2005; Wortmann et al., 2007). Viewed as the slope in a ${\delta^{18}O}_{{\mathrm{SO}}_{4}}$ vs. ${\delta^{34}S}_{{\mathrm{SO}}_{4}}$ plot, relatively steep slopes have been shown to characterize relatively slow net MSR rates (as low as 10^−12^ mol cm^−3^ year^−1^; representing higher re‐oxidation) while moderate slopes characterize relatively fast net MSR rates (maximum of 5 × 10^−4^ mol cm^−3^ year^−1^; limited re‐oxidation) (Antler et al., 2013, 2015). Other processes are known to have an effect on the ${\delta^{18}O}_{{\mathrm{SO}}_{4}}$ vs. ${\delta^{34}S}_{{\mathrm{SO}}_{4}}$ plot such as the sulfide and pyrite oxidation (Balci et al., 2007) and disproportionation (Böttcher et al., 2001, 2005); however, their effect should only decrease the relative estimated calculated rates of MSR. Additionally, it was shown that when the sulfate reduction rates are high (i.e., the ${\delta^{18}O}_{{\mathrm{SO}}_{4}}$ vs. ${\delta^{34}S}_{{\mathrm{SO}}_{4}}$ slope is low), the effect of transport such as diffusion and sedimentation is negligible on the measured slope (Fotherby et al., 2020). Such a plot can be adapted to the form of (${\delta^{18}O}_{{\mathrm{SO}}_{4}}$‐ ${\delta^{18}O}_{{\mathrm{SO}}_{4},0}$) vs. (${\delta^{34}S}_{{\mathrm{SO}}_{4}}$‐ ${\delta^{34}S}_{{\mathrm{SO}}_{4},0}$), where ${\delta^{18}O}_{{\mathrm{SO}}_{4},0}$ and ${\delta^{34}S}_{{\mathrm{SO}}_{4},0}$ are the initial SO_4_^2−^ isotope values, thereby negating the “source effect” and allowing data with a linear relationship from various aquatic and subsurface environments beyond the Dead Sea to be compared (e.g., lakes, seas, estuaries, and salt marshes) (Figure 4).

Pore fluid SO_4_^2−^ from the interval of interest has a calculated slope of 0.26 for (${\delta^{18}O}_{{\mathrm{SO}}_{4\;}}$‐ ${\delta^{18}O}_{{\mathrm{SO}}_{4},0}$) vs. (${\delta^{34}S}_{{\mathrm{SO}}_{4}}$ ‐ ${\delta^{34}S}_{{\mathrm{SO}}_{4},0}$), which is low compared to other aquatic systems in the region and suggests that MSR was comparably faster (Figure 4: circle markers; Supplementary Figure S4). Pore fluids from deep Mediterranean Sea sediments plot on a steeper slope of ~1.1 (Antler et al., 2015; Figure 4: empty triangle markers), while methane cold seeps in the Eastern Mediterranean (Rubin‐Blum et al., 2014; Figure 4: +markers), Lake Kinneret (Sea of Galilee) hypolimnion (Knossow et al., 2015; Figure 4: empty diamond markers), and the Yarqon estuary (Antler et al., 2013; Figure 4: square markers) have slopes in the range of 0.7 to 0.3 (Figure 4); all hinting at relatively slower net MSR rates compared to the Dead Sea interval of interest.

Stable ${\delta^{34}S}_{{\mathrm{SO}}_{4}}$ and ${\delta^{18}O}_{{\mathrm{SO}}_{4}}$ from primary gypsum layers found in the Dead Sea sedimentary record were also used to investigate rates of MSR using a similar isotope plot given that the effect of isotope fractionation during gypsum precipitation has a negligible effect on the slope (Torfstein & Turchyn, 2017). Primary gypsum deposits dating ca. 61–14.5 ka from the last glacial Dead Sea (Lake Lisan) captured the isotopic composition of lake water SO_4_^2−^ with relatively minor isotopic fractionation during precipitation. These deposits have ^34^S‐ and ^18^O‐enriched stable isotope signatures (${\delta^{34}S}_{Ca{\mathrm{SO}}_{4}∙2H_{2}O}$ and ${\delta^{18}O}_{Ca{\mathrm{SO}}_{4}∙2H_{2}O}$) suggesting that MSR occurred over extended period of time in the last glacial Dead Sea water column (Torfstein et al., 2005, 2008; Torfstein & Turchyn, 2017). On a multi‐millennial timescale, Lake Lisan was characterized by ongoing and increasing lake levels (i.e., positive net water balance), which reached a high stand around 31–17.4 ka (~Last Glacial Maximum; Torfstein et al., 2013). Primary gypsum layers from the Lisan Fm. have steeper ${\delta^{34}S}_{{\mathrm{SO}}_{4}}$ vs. ${\delta^{18}O}_{{\mathrm{SO}}_{4}}$ slopes than all the modern regional sites (~1.1 to 2; Figure 4) and were suggested to have recorded a very slow net MSR rate in the stratified (paleo) water column. Comparatively, gypsum from the preceding interglacial Amora Fm. has a slope of ~0.55 (Torfstein & Turchyn, 2017).

The slope value of 0.26 from the interval of interest is comparable to the slope from areas of intensified MSR such as the East Anglian salt marshes (black filled triangles; the slope of 0.24) (Antler et al., 2019). It may also be comparable to modern hypersaline groundwater around the shores of the western Dead Sea banks (Avrahamov et al., 2014). It is reasonable to assume that rates of MSR may have been high; in the Dead Sea, microbial mats from underwater emerging springs close to the western shores have large spatial heterogeneity in sulfate reduction rates, with values up to 10 nmol cm^−3^ day^−1^ (4 × 10^−6^ mol cm^−3^ year^−1^) detected in saline springs (Häusler et al., 2014).

In this model for interpretation of the evolution of ${\delta^{34}S}_{{\mathrm{SO}}_{4}}$ vs. ${\delta^{18}O}_{{\mathrm{SO}}_{4}}$, the lower the slopes are the more they reflect fast (intensified) MSR rates which in turn may be dependent on the type of organic substrate used by the microbial community (Antler et al., 2017). Sulfate reduction coupled to the anaerobic oxidation of methane (AOM‐SR; Equation 6) often exhibits a fast rate of MSR and correspondingly moderate ${\delta^{34}S}_{{\mathrm{SO}}_{4}}$ vs. ${\delta^{18}O}_{{\mathrm{SO}}_{4}}$ slopes are often detected (Antler et al., 2013, 2015):

(6) $${{\mathrm{SO}}_{4}}^{2‐}+{\mathrm{CH}}_{4}\;\to {\mathrm{HCO}}_{3}^{‐}+{\mathrm{HS}}^{‐}+H_{2}O$$

**FIGURE 5:**
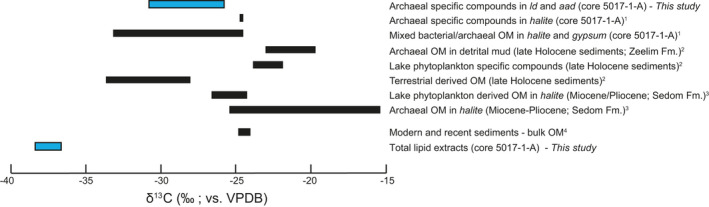
Range of measured ${\delta^{13}C}_{\mathrm{Org}}$ (‰; vs. VPDB) in the Dead Sea and sediments. References for data within figure: Thomas, Ionescu, Ariztegui & DSDDP Scientific Team (2015); Oldenburg et al. (2000); Grice et al. (1998); Nissenbaum et al. (1972)

Evidence for AOM‐SR has been previously found in hypersaline groundwater from the western margin of the Dead Sea (Avrahamov et al., 2014) but, as of yet, not in the hypolimnion environment. During AOM‐SR (Equation 6), the addition of HCO_3_^−^ derived from methane leads to a more pronounced decrease in the $\delta^{13}C$ of the DIC reservoir compared to oxidation of OM via organoclastic sulfate reduction. In the pore fluid samples from the interval of interest, there is an increase of DIC from the mean value of 0.6 mmol Kg^−1^ to between 2.1 and 1.9 mmol Kg^−1^, coupled with a drop in ${\delta^{13}C}_{\mathrm{DIC}}$ from mean value of −5.4‰ to between −16‰ and −12‰ (Figure 2g). These ${\delta^{13}C}_{\mathrm{DIC}}$ values would be indicative of extensive AOM‐SR had they been more negative. Partial organic carbon oxidation into a relatively large and isotopically ^13^C‐enriched DIC pool may have occurred. An isotope mass balance calculation can be used to determine the $\delta^{13}C$ of OM oxidized. Using averages of pre‐peak ${\delta^{13}C}_{\mathrm{DIC}}\;\&\;DIC$ and peak ${\delta^{13}C}_{\mathrm{DIC}}\;\&\;DIC$ values for this mass balance calculation must also account for lake dilution (as expressed by Mg^2+^) and an unknown DIC enrichment, which adds uncertainty. Thus, a mass balance calculation that calculates the $\delta^{13}C$ of OM oxidized within the peak ${\delta^{13}C}_{\mathrm{DIC}}$ depth interval based on discrete measurements is used (see methods; Equation 5), providing a ${\delta^{13}C}_{\mathrm{OM}}$ value of −47‰. This estimate is lower than the range of primary derived OM in the Dead Sea (Figure 5) and closer to that of CH_4_ (−40‰) found in the Dead Sea hypersaline groundwater along the western margins (Avrahamov et al., 2014). However, this calculation is somewhat speculative as it is based only on two discrete measurements; had there been more measurements in this interval the calculated ${\delta^{13}C}_{\mathrm{OM}}$ would give more confidence. Furthermore, it should be noted that the depth of maximum ${\delta^{18}O}_{{\mathrm{SO}}_{4}}$ and ${\delta^{34}S}_{{\mathrm{SO}}_{4}}$ values overlap only partly with the depths of the lowest ${\delta^{13}C}_{\mathrm{DIC}}$ peak (Figure 2g). This is due to the low vertical resolution of pore fluid sampling and limitation in volumes of extracted pore fluid that did not allow measuring ${\delta^{18}O}_{{\mathrm{SO}}_{4}}$ and ${\delta^{34}S}_{{\mathrm{SO}}_{4}}$ at the most depleted ${\delta^{13}C}_{\mathrm{DIC}}$ anomaly.

Lipid biomarkers from sediments can be used to detect evidence of methanogenesis, the presence of anaerobic methanotrophic archaea (ANME), and syntrophic sulfate‐reducing bacteria (SRB) involved in AOM (Blumenberg et al., 2004). Nonisoprenoid macrocyclic glycerol diethers (a–g in Figure 3d; S17, S18, S19) support the existence of bacterial communities potentially involved in the S‐cycle and have been detected in hypersaline sulfidic sediments (Baudrand et al., 2010) or environments with AOM (van Dongen et al., 2007). Macrocyclic archaeol (Figure 3d; S17) has been identified in the methanogenic archaea *Methanococcus janaschii* isolated from a deep‐sea hydrothermal vent (Comita & Gagosian, 1983). Phytane detected in S17 and S18 (Figure 3c) can constitute a marker of methanogenic archaeal communities (Risatti et al., 1984; Schouten et al., 1997; Tornabene et al., 1979), although this compound has also been attributed to halophilic archaea in hypersaline environments and, in particular, the Dead Sea realm (Anderson et al., 1977). It is noteworthy that crocetane and pentamethylicosane (PMI), which can be strong indicators of AOM (Pancost et al., 2000) were not detected in the investigated Dead Sea samples. Finally, sn‐2‐hydroxyarchaeol has been described as a characteristic lipid biomarker of ANMEs (Figure 3d; S17, S18, S19) (Hinrichs et al., 2000). The combination of archaeol and hydroxyarchaeol has been proposed as a biomarker signature specific to some CH_4_ seeps (Blumenberg et al., 2004; Knittel & Boetius, 2009), where anaerobic CH_4_ oxidizers and methanogenic archaea can co‐exist and produce a similar set of biomarkers (Schouten et al., 1997). Unfortunately, the low amount of hydroxyarchaeol in the investigated samples did not allow for the measurement of its $\delta^{13}C$ values that could have informed us on the occurrence of AOM. On the other hand, the range of $\delta^{13}C$ values measured for Dead Sea archaeol (between −30 and −25‰) indicates an origin of this compound different from ANME, and its abundance along with that of extended archaeol (Figure 3d) rather supports halophilic archaea of the Haloarchaea class as a major biological source of archaeol (Vandier et al., 2021). These organisms classically dominate in the Dead Sea modern and ancient waters (Bodaker et al., 2010; Thomas & Ariztegui, 2019), and sediments (Grice et al., 1998; Thomas et al., 2015, Thomas, Grossi, et al., 2019). The set of biomarkers and their carbon stable isotope composition present in this interval therefore support a hypersaline environment dominated by halophilic archaea. The slightly depleted $\delta^{13}C$ values measured for the total lipid extracts (ranging between −38‰ and −36‰) compared to that of archaeol still potentially argue for the presence of methane in this environment, without indicating AOM‐SR.

### 3.3 Autochthonous vs. allochthonous sources of OM

The availability of OM and its lability are important factors determining the rate of MSR in saline environments (marine: Schubert et al. (2000); lacustrine: Glombitza et al., 2013). In the modern Dead Sea, there is a low OM influx from the water column to the sediment (low benthic oxygen uptake of 0.46 mmol m^−2^ day^−1^), a result of the general lack of primary production and low input of terrestrial organic carbon to the lake (Häusler et al., 2014). It has previously been suggested that the slow rate of MSR in sediments of the Dead Sea is due to the lack of availability and/or OM quality (Häusler et al., 2014; Thomas et al., 2016).

Measured total organic carbon (TOC) content in the interval of interest are below 1 wt % (Thomas et al., 2015; Supplementary Figure S5) and comparable over the Holocene sedimentary section. Given no significant increase of TOC (%) suggests that the preservation of OM in the deep Dead Sea was not dissimilar over wet and dry climate intervals during the Holocene. Thus, observed OM compositional changes in the Holocene sediments are likely to reflect allochthonous and/or autochthonous derived OM in the Dead Sea. Lipid biomarkers can be used to estimate the presence of allochthonous and autochthonous derived OM. The presence of *n*‐alkanes with an odd‐over‐even carbon number predominance centered at C_29_ and C_31_ in all sediment samples investigated (S17, S18, S19, S21; Figure 3c) infers a proportion of allochthonous derived OM, probably from plant waxes (Meyers, & Ishiwatari, 1993). This confirms findings by Oldenburg et al. (2000) defining terrestrial (allochthonous) OM as an important source to the Dead Sea sediment. However, in the sediment interval of interest (S17 and S18), the presence of *n*‐C_17_ alkane points to an additional contribution from cyanobacteria (Sachse et al., 2006) or phytoplankton to the bulk OM, that appears together with evidence of increased freshwater influx and intensified MSR. This lipid biomarker is not found in the S19 and S20 samples from the halite‐rich interval below. The presence of phytane (as a degradation product of the chlorophyll side chain in anoxic environments) may also be indicative of cyanobacterial or phytoplankton source (*Dunaliella*?) for organic matter to the Dead Sea sediments, although as degradation occurs in more mature sediments its origin from Haloarchaea in this setting is more likely. A large range of molecular C:N ratio values (from 3.5 to 19.8) were found in this depth interval, which suggests that both prokaryotic/algal aquatic OM and plant‐derived OM from terrestrial/allochthonous sources reached the hypolimnion (Thomas et al., 2015; Supplementary Figure S5). Being labile, autochthonous derived OM may have provided an OM source fueling intensified organoclastic type MSR (Ebert et al., 2020; Thomas et al., 2016) or alternatively led to enhanced methane production that potentially fueled AOM‐SR.

### 3.4 Regional climate driver of MSR in the Dead Sea

**FIGURE 6:**
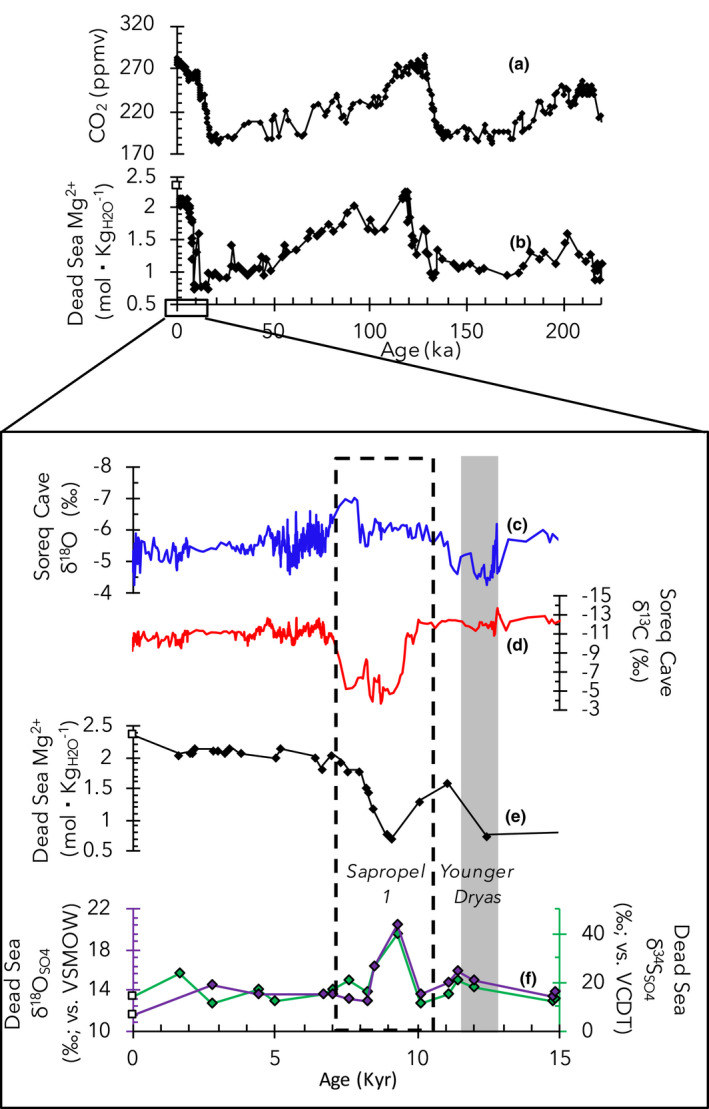
Paleoclimate and Dead Sea time‐series records. From top to bottom (a) Compiled atmospheric CO_2_ record from Antarctic ice cores (Monnin et al., 2001; Pépin et al., 2001; Petit et al., 1999); (b) Dead Sea pore fluid Mg^2+^ record from 5017‐1‐A (correlation after Levy et al., 2017). Focusing on the last 15 kyr's: Soreq speleothem records of (c) $\delta^{18}O$ (blue) and (d) $\delta^{13}C$ (red) (Bar‐Matthews et al., 2003; Grant et al., 2012); (e) Dead Sea Mg^2+^ concentrations (Levy et al., 2017); (f) Dead Sea pore fluid ${\delta^{34}S}_{{\mathrm{SO}}_{4}}$ (green) and ${\delta^{18}O}_{{\mathrm{SO}}_{4}}$ (purple) isotope values. Modern 2013 composition of Dead Sea at 100‐m depth (${\delta^{34}S}_{{\mathrm{SO}}_{4}}$ = 14.1‰; ${\delta^{18}O}_{{\mathrm{SO}}_{4}}$ = 11.4‰) shown as white squares for comparison (Levy et al., 2018, 2019)

Intensified MSR and changes in lipid biomarker distribution were coeval with positive net water balance in the Dead Sea, ultimately resulting from wet regional hydroclimate conditions. Global climate change on a multi‐millennial scale inferred from decreasing and increasing CO_2_ concentrations in Antarctic ice‐core records emphasize the natural oscillation between glacial and interglacial periods, respectively (Figure 6a; Monnin et al., 2001; Pépin et al., 2001; Petit et al., 1999). Illustrating the control of global climate on the net water balance of the terminal Dead Sea, similar trends are found in the deep Dead Sea composition as shown by decreasing (positive water balance) and increasing (negative water balance) conservative Mg^2+^ concentrations from pore fluids from 5017‐1‐A (Figure 6b; Levy et al., 2017). Perturbing these long‐term global climate patterns in the Mg^2+^ record is a dilution event correlative to wet/humid conditions occurring locally in the Eastern Mediterranean region during the early Holocene at ca. 9500–8300 years BP (Figure 6b and e).

Increased rainfall in the Eastern Mediterranean during the early Holocene is evident in speleothem records from Jeita cave (western Lebanon) (Verheyden et al., 2008), Soreq cave (central Israel) (Bar‐Matthews et al., 2003), and other regional paleoclimate records (Cheng et al., 2015; Robinson et al., 2006). Calcium carbonate speleothem stable oxygen ($\delta^{18}O$) and carbon ($\delta^{13}C$) isotope ratios from the Soreq cave provide high‐resolution paleoclimate records of local rainfall conditions in relative proximity to the Dead Sea (found ~50 km east; Figure 1) and suggest that the early Holocene was marked by increased rainfall relative to the preceding drier onset of Holocene/post‐Younger Dryas (YD) and following mid and late Holocene (Bar‐Matthews et al., 2003). Speleothem $\delta^{18}O$ values (marked by dashed line area in Figure 6) range between −6‰ and −7‰, the lowest values found in the Holocene record (Bar‐Matthews et al., 2003). Anomalously high $\delta^{13}C$ values of −4‰ to −5‰ are found at ca. 9 to 8 ka in comparison to the typical ~ −13‰ to −10‰ and were suggested to be the result of host rock chemical weathering following severe floods. Further evidence for increased rainfall during this time was derived from the reconstruction of maximum cave paleopool levels (Bar‐Matthews et al., 2003). During the middle and late Holocene, the speleothem records suggest a return to relatively dry climate conditions and are corroborated by increasing Mg^2+^ concentrations in pore fluids and a succession of layered halite in the Dead Sea ICDP sediment cores (Figure 6c, d, and e).

The Dead Sea received most of its freshwater runoff from the Jordan River via the northern catchment during the early Holocene (Palchan et al., 2019). Westwards beyond the Dead Sea and Soreq cave in the adjacent Mediterranean Sea and central Mediterranean region, microbial ecology responses to hydroclimate changes occurred (Ariztegui et al., 2000). In both the Mediterranean Sea and the Dead Sea freshwater‐derived and less saline buoyant surface water layers formed. Increased freshwater influx to the Mediterranean Sea predominantly from the River Nile and derived from African monsoon rainfall, led to the formation of organic‐rich sapropel layer S1, spanning from 10,500 to 6100 years BP (Grant et al., 2016). Stratification in parts of the Mediterranean Sea resulted in eutrophication, oxygen‐poor lower water, and benthic azoic conditions, which culminated in the deposition of the sulfide and C_organic_ rich, sapropel layer 1 (S1) (e.g., Almogi‐Labin et al., 2009; Rohling et al., 2015). In the epilimnion of the stratified Dead Sea primary productivity occurred as evident by the presence of *n*‐C_17_ alkane, while in the deep Dead Sea (hypolimnion) intensified MSR occurred at/below the sediment‐water interface (Figure 6f). Collectively, the marine and lacustrine evidence suggest that the microbial ecology in both the surface and deep Mediterranean Sea and Dead Sea were independently responding to the increased influx of freshwater during the early Holocene.

## 4 SUMMARY

The present study provides insight into the microbial response in the Dead Sea to positive net water balance caused by enhanced regional hydroclimate activity between ca. 9500–8300 years BP. This was done by measuring the Dead Sea drilled ICDP core sediments pore fluid concentrations and stable S, O, and C isotopes, combined with sediment lipid biomarkers. Positive lake net water balance was accompanied by depositional changes and surface water dilution, which facilitated enhanced microbial processes in both the surface and deep Dead Sea. Analogous to modern‐day spontaneous blooms of the primary producer *Dunaliella parv*a and halophilic archaea following increased freshwater runoff, lipid biomarkers archived from the deep sediments suggest the onset of upper water column productivity following positive lake net water balance. In the anoxic hypolimnion and/or its bottom sediments there was a microbial response manifested as intensified microbial sulfate reduction.

## CONFLICT OF INTEREST

None.

## Supporting information

Fig S1‐S5

## Data Availability

The data that support the findings of this study are available from the corresponding author upon reasonable request.
