# Supplementary material for: Intensified microbial sulfate reduction in the deep Dead Sea during the early Holocene Mediterranean sapropel 1 deposition
Source: Geobiology. 2022 Apr 5;20(4):518–32. doi: 10.1111/gbi.12493 (PMC9325388; doi:10.1111/gbi.12493)
Supplement: Supplementary file 1 — Fig S1‐S5 [file GBI-20-518-s001.docx]

**Supplementary figures for Levy et al.**

**Supplementary Fig. S1: 5017-1-A Pore fluid and halite inclusion concentration and ratio profiles (after Levy et al., 2017 and Kiro et al., 2017)**


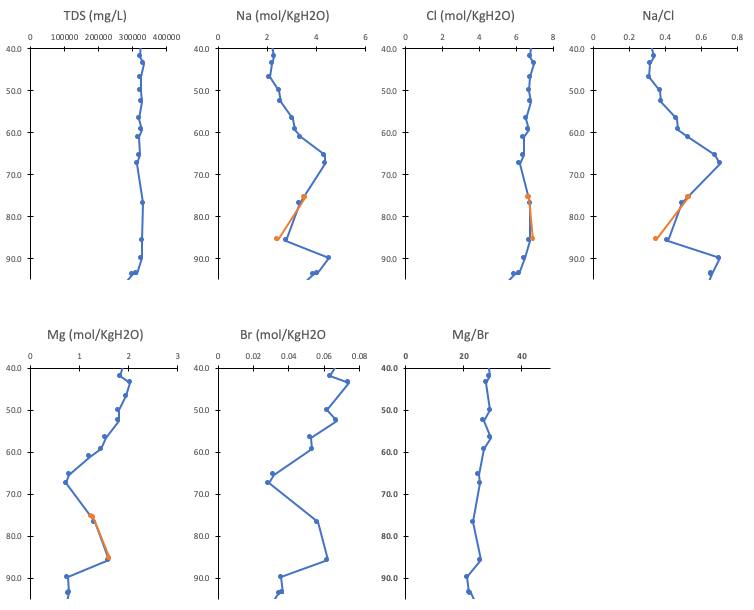


Geochemical profiles of pore fluids (blue circles) and fluid inclusions (orange circles) from Holocene interval of core 5017-1-A. (a) TDS (mg·L^-1^); (b) Na+ (mol·Kg(H2O) ^-1^); (c) Cl- (mol·Kg(H2O) ^-1^); (d) Na/Cl; (e) Mg (mol·Kg(H2O) ^-1^); (f) Br (mol·Kg(H2O) ^-1^); (g) Mg/Br. The pore fluid concentrations were compared with concentrations in fluid inclusion in the primary halite samples measured using cryo-SEM-EDS technique as described in Kiro et al. (2017).

**Supplementary** **Fig. S2: Mass spectra of Macrocyclic Glycerol Diethers (MGD) compounds (a-g) and macroarchaeol (h) identified in sample S17.**

**Supplementary Fig. S3: Proposed structures of macrocyclic glycerol diether compounds (a-g) and macrocyclic archaeol (h)**


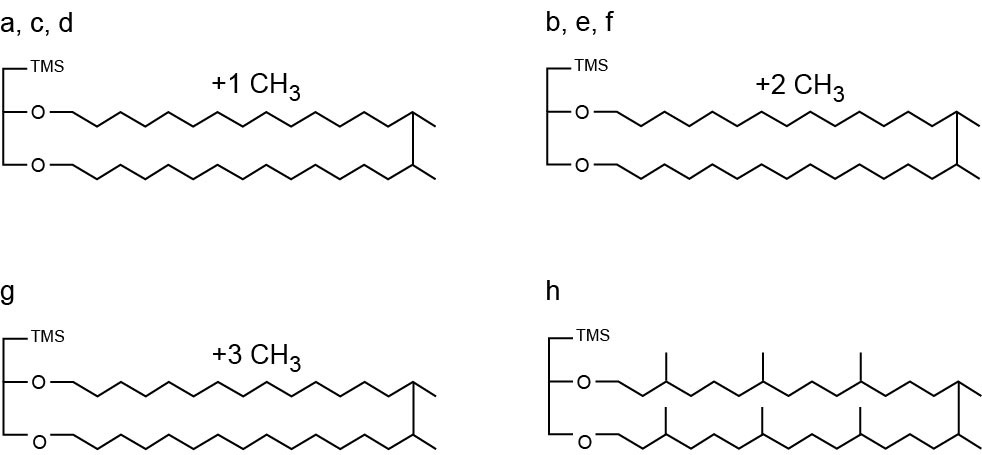


**Supplementary Fig. S4: δ^18^O_SO4_ vs. δ^34^S_SO4_ of early & middle Holocene Dead Sea pore fluids**

Blue circles: Early and middle Holocene δ^18^O_SO4_ and δ^34^S_SO4_ in pore fluids. Red triangle: average isotope composition of pore fluid samples with isotope values showing no apparent/significant microbial sulfate reduction (δ^34^S _SO4_ < 20‰), δ^34^S _SO4,0_ = 15.0‰ and δ^18^O_SO4,0_ = 13.9‰. The linear trend (yellow dotted line) includes this value with the two isotope values > δ^34^S=25‰ from the depth of interest (Manuscript Fig. 2); Black cross: 2013 Dead Sea 100m water column depth sulfate isotope composition: δ^34^S _SO4,0_ = 14.1‰ and δ^18^O_SO4,0_ = 11.4‰; Green square: Modern (2012) Dead Sea SO_4_^2-^ - δ^34^S _SO4,0_ = 15.9‰ and δ^18^O_SO4,0_ = 13.65‰ (from Avrahamov et al., 2014).

**Supplementary Fig. S5: Depth profiles of TOC (%) and molecular C/N ratio of OM in deep Dead Sea core 5017-1-A (after Thomas et al., 2015)**


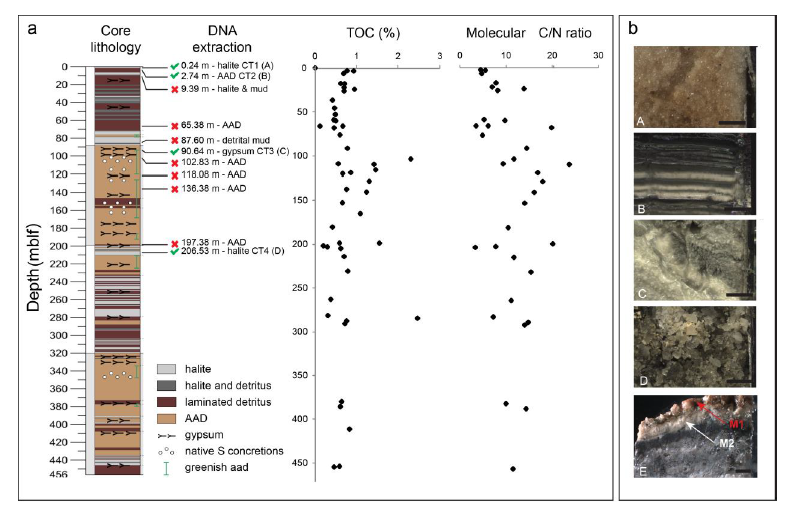


Adapted from Thomas et al., (2015). Left to right: lithological description of the studied core modified from Neugebauer et al. (2014), depths where DNA extraction had been attempted, TOC (%) and C/N profiles. In turquoise the depth interval of interest in this paper.
